# Engineering of *Escherichia coli* for Co-Production of Lignocellulosic Ethanol and Poly(3-hydroxybutyrate)

**DOI:** 10.3390/microorganisms14030537

**Published:** 2026-02-26

**Authors:** Nguyen Luan Luu, Yin-Zhou Liu, Doan Thanh Ta, Chung-Jen Chiang, Yun-Peng Chao

**Affiliations:** 1 Department of Chemical Engineering, Feng Chia University, No. 100, Wenhwa Road, Taichung 40724, Taiwan; luunguyenluan@gmail.com (N.L.L.);; 2 Department of Medical Laboratory Science and Biotechnology, China Medical University, No. 91, Xueshi Road, Taichung 404328, Taiwan; 3 Department of Medical Research, China Medical University Hospital, No. 2, Yude Road, Taichung 404327, Taiwan

**Keywords:** lignocellulosic ethanol, poly(3-hydroxybutyrate), rice straw hydrolysate, metabolic engineering

## Abstract

Bioethanol is an alternative energy source to fossil fuels and can serve as a raw material for the production of sustainable aviation fuel. Poly(3-hydroxybutyrate) (PHB) is a biodegradable plastic with the potential to replace petrochemical plastics. Lignocellulose has a renewable and eco-friendly nature, and it is a key factor in determining the environmental impact of bioethanol and PHB. In this study, we addressed this issue by developing *Escherichia coli* for the co-production of bioethanol and PHB from rice straw hydrolysate (RSH). Metabolic evolution was employed to enhance ethanol tolerance in the ethanologenic *E. coli* strain. To mitigate the toxicity of RSH, the strain was modified by rewiring the pentose phosphate pathway and subsequently subjected to metabolic evolution. The strain was further reshaped by reprogramming xylose metabolism and recruiting the PHB synthesis pathway. As a result, the engineered strain simultaneously utilized glucose and xylose while producing 19.8 g/L of bioethanol and 3.5 g/L of PHB in 30 h. The bioethanol yield and the PHB content account for 0.40 g/g and 38% of dry cell weight, respectively. Overall, it indicates the potential application of this developed strain in lignocellulosic biorefineries.

## 1. Introduction

Fossil fuels are neither renewable nor environmentally friendly, yet they provide the energy that currently sustains our social activities. The consumption of these conventional fuels increases significantly with the continued growth of the global population and industrialization, resulting in massive CO_2_ emissions [1]. This consequently causes drastic climate change, which has worsened human welfare and quality of life. Therefore, it is imperative to implement eco-friendly energy. Biofuels are recognized as a potential candidate due to their sustainability and environmental compatibility [2]. Bioethanol is of industrial interest and has a superior octane number and combustion efficiency than gasoline [3]. The liquid fuel, consisting of gasoline blended with bioethanol, is widely used in the transportation sector today. Jet fuel consumption in the aviation industry has grown significantly, accounting for 21% of global energy demand in the transportation sector [4]. Achieving a 50% reduction in CO_2_ emissions by 2050 is hardly feasible without the availability of green jet fuel. Biojet fuel obtained from the alcohol-to-jet (ATJ) process appears to meet this goal. The processing of bio-based alcohols in ATJ has been certified by the American Society of Testing and Materials [5], thereby rendering lignocellulosic ethanol a viable option.

The global bioethanol market volume is rapidly growing, driven by increasing demand for transport fuels, and has reached over 140 billion liters [6]. The USA and Brazil are two major suppliers, accounting for approximately 84% of the global bioethanol market. They primarily exploit microbial fermentation using corn starch and sugarcane to produce first-generation (1G) bioethanol [7]. However, the exploitation of sugars derived from food crops inevitably provokes controversy in the food-and-feed debate. The development of the 1G bioethanol process is further hindered by critical challenges, including sustainability concerns, land-use restrictions on farming, and the availability of clean water [8]. The problems associated with 1G bioethanol can be addressed by second-generation (2G) bioethanol, which is produced through fermentation using non-food crops and crop waste residues as feedstocks [9].

Plastic consumables derived from fossil fuels are ubiquitous in our daily lives. Their economic value increases significantly as the evolving needs of industrialization emerge [10]. Nevertheless, these petrochemicals have a high carbon footprint and, in particular, persist in the environment. This poses a severe threat to the ecology and human health [11,12]. Polyhydroxyalkanoates (PHAs) naturally occur in microbes and are biodegradable plastics with the potential to replace petrochemical plastics [13]. Poly(3-hydroxybutyrate) (PHB) is a member of the PHA family and has garnered significant interest in the industry [14]. High production costs continue to hinder the commercialization of PHB, despite numerous studies on the development of microbial production processes for PHB.

Lignocellulosic biomass is the most abundant natural resource and appears to be a cost-effective and renewable feedstock [15]. Lignocellulose comprises cellulose and hemicellulose and produces glucose and xylose after hydrolysis. Yeast and bacteria have been engineered for fermentative production of lignocellulosic ethanol from lignocellulosic hydrolyates [16,17]. Ethanologenic bacteria generally display superior performance to yeast. It has been reported that the fermentation efficiency of rice straw-based lignocellulosic ethanol significantly contributes to environmental sustainability, as assessed by life-cycle assessment [18]. The cost of carbon sources accounts for half of the production cost in PHB fermentation [19]. Apparently, an inexpensive and sustainable carbon source is a key to ensuring the economic viability of bioethanol and PHB. The co-production of biochemicals with ethanol is recognized as a promising approach to enhance the efficiency of lignocellulosic biorefineries [20]. Known for its biotechnology-friendly nature, *Escherichia coli* is cultivable in cost-effective media and utilizes various sugars. Accordingly, the current study aimed to develop *E. coli* for the production of bioethanol and PHB based on rice straw hydrolysate (RSH). This was achieved through adaptive evolution and genetic modification of *E. coli* to confer the desired trait. To the best of our knowledge, this is the first study reporting the co-production of lignocellulosic ethanol and PHB in *E. coli*.

## 2. Materials and Methods

### 2.1. Bacterial Culturing

Cell growth was measured turbidimetrically with the UV-1800 spectrophotometer (Shimadzu, Kyoto, Japan) at 550 nm (OD_550_). The cell biomass at OD_550_ of 1 was equivalent to 0.33 mg dry cell weight (DCW) per mL. Unless stated otherwise, the ethanol fermentation was carried out under oxygen-limited conditions. Engineered *E. coli* strains were grown overnight on LB medium. Following centrifugation, harvested cells were seeded into Erlenmeyer flasks (125 mL) containing diluted RSH (50 mL) as indicated. RSH was obtained from the Institute of Nuclear Energy Research (Taoyuan, Taiwan) and mainly contained around 57 g/L glucose and 15 g/L xylose. RSH was diluted with water for use as needed. The fermentation was initiated with an initial cell density (ICD) at OD_550_ of 0.2 and conducted in an orbital shaker set at 37 °C and 120 rpm. Aerobic culturing was performed by seeding cells into Erlenmeyer flasks containing 20 mL of RSH and corn steep liquor (CSL) (Tairoun Production Co., Yunlin, Taiwan). The administered dosage of CSL was 3% (*v*/*v*). The culture was maintained at 37 °C with shaking at 200 rpm. The bioethanol yield (*g*/*g*) is calculated based on the total consumption of glucose and xylose.

### 2.2. Adaptive Evolution

*E. coli* strain was evolved for tolerance of ethanol and RSH. This was first carried out using a serial subculture of cells with an increasing level of ethanol. The strain was cultured in the Erlenmeyer flask (125 mL) containing LB medium (20 mL) supplemented with 40 g/L glucose and 3% ethanol. The ICD at OD_550_ of 0.1 was used to initiate cell evolution. The culture was incubated at 37 °C with brief shaking for 24 h, then transferred to fresh medium for a new cycle of evolution. Ethanol concentration increased as cell density rose in successive transfers. Finally, the ethanol-tolerant strain that grew well was isolated. In addition, the RSH-resistant strain was evolved in a similar manner. The strain was grown on diluted RSH, followed by serial transfers at increasing RSH concentrations. The evolved strain was consequently isolated.

### 2.3. Strain Development

Table 1 summarizes the strains, plasmids, and primers used herein. In this study, the BL21p strain was employed for further development [21]. Expression of *zwf* and *pgl* in the BL21-PP strain was enhanced using the reported method [22]. In brief, the passenger DNA was amplified from either plasmid pPR-zwf or pSPL-atoD. Following electroporation, each linear DNA was integrated into the genome of the host strain with the aid of λ Red. The genetic construction resulted in the fusion of λP_L_ promoter with endogenous *zwf* and *pgl*. Moreover, the *xylAB* operon in the strain was fused to the *trc* promoter (P*trc*). This was carried out by PCR to amplify LE*-*kan*-RE*-P*trc* with Xyl-1 and Xyl-2 primers, the upstream of *xylAB* operon with Xyl-3 and Xyl-4 primers, and the structural gene of *xylAB* operon with Xyl-5 and Xyl-6 primers. The overlap extension PCR was performed by mixing three PCR DNAs at a 1:1:1 weight ratio. After 15 cycles of PCR, Xyl-1 and Xyl-6 primers were added to the reaction mixture. PCR was continued for an additional 20 cycles to yield a DNA cassette comprising LE*-*kan*-RE*-P*trc*, flanked by two homology arms. The DNA cassette was used for genomic insertion through λ Red-mediated homologous recombination. The inserted antibiotic marker associated with each integration event was later removed by Cre [23]. Additionally, the PHB-producing strain was developed by integrating the *Cupriavidus necator phaCAB* operon. This was carried out using the pLam-Pha and pPhi80-Pha plasmids, which contain the *phaCAB* operon under the control of λP_L_, according to the reported protocol [24]. This resulted in the strain carrying λP_L_-*phaCAB* at the λ *attB* and ϕ80*attB* sites, respectively.

### 2.4. Analytical Methods

Analytical methods were performed as described in the reported protocol [21,24]. High-performance liquid chromatography (HPLC) equipped with a refractive index detector RID-10A (Shimadzu, Kyoto, Japan) was used to measure glucose and xylose. The analysis was conducted using the ICSep ICE-ION-300 column (Transgenomic, Omaha, NE, USA) with the mobile phase (0.0085 N sulfuric acid) at 0.4 mL/min. Ethanol was determined using a Porapak Q 80/100 column (Merck Supelco, Darmstadt, Germany) installed in a gas chromatograph Trace 1300 (Thermo Fisher, Waltham, MA, USA). Samples were eluted for analysis using a carrier gas consisting of air, nitrogen, and hydrogen at the predetermined gauge pressure. To analyze PHB, harvested cells were dried in an oven at 55 °C overnight. The dried cells were weighed and treated with 2N NaOH at 95 °C for 1 h. After adding 2N H_2_SO_4_, crotonic acid derived from PHB was measured by HPLC with the Aminex HPX-87H ion exclusion column (Bio-Rad, Hercules, CA, USA) with the mobile phase (0.0085 N sulfuric acid) at 0.3 mL/min. A UV detector at 210 nm was used to analyze the eluent.

## 3. Results and Discussions

### 3.1. Development of the Ethanologenic Strain

**Figure 1:**
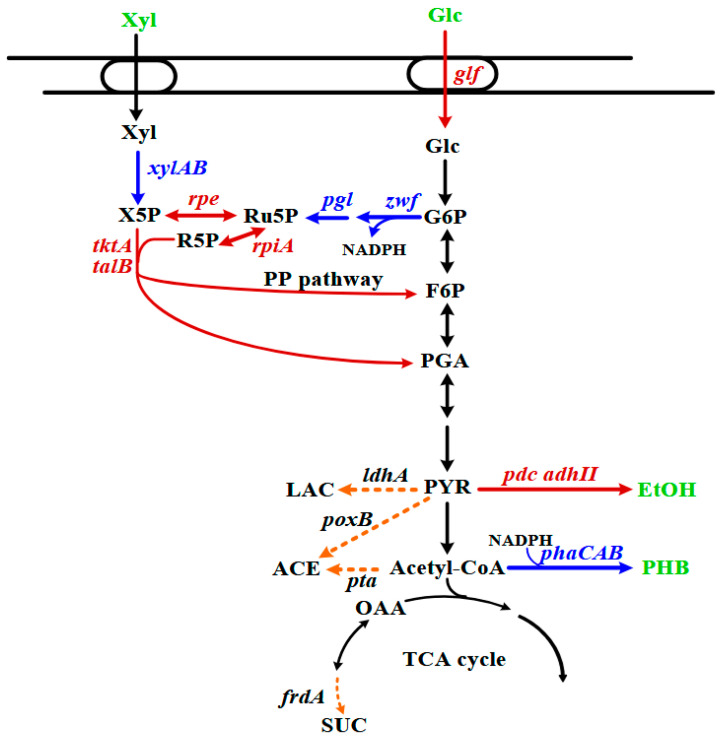
The central metabolic pathway leading to ethanol and PHB in *E. coli.* The ethanol-producing strain harbored engineered metabolic pathways (red) and impaired reaction routes (dotted lines). This strain was further reprogrammed by manipulating metabolic pathways, as indicated in blue. The metabolic pathway involves genes of interest as follows: *adhII* (*Z. mobilis*), alcohol dehydrogenase; *frdA*, fumarate reductase; *glf* (*Z. mobilis*), glucose facilitator; *ldhA*, lactate dehydrogenase; *pdc* (*Z. mobilis*), pyruvate decarboxylase; *pgl*, lactonase; *phaA*, β-ketothiolase; *phaB*, acetoacetyl-CoA reductase; *phaC*, PHB synthase; *poxB*, pyruvate oxidase; *pta*, phosphate acetyltransferase; *rpe*, ribulose-5-phosphate 3-epimerase; *rpiA*, ribose-5-phosphate isomerase A; *talB*, transaldolase B; *tktA*, transketolase A; *xylA*, xylose isomerase; *xylB*; xylulokinase; *zwf*, glucose 6-phosphate dehydrogenase. Abbreviations: ACE, acetate; EtOH, ethanol; F6P, fructose 6-phosphate; Glc, glucose; G6P, glucose 6-phosphate; LAC, lactate; OAA, oxaloacetate; PGA, 3-phosphoglyceraldehyde; PYR, pyruvate; Ru5P, ribulose 5-phosphate; R5P, ribose 5-phosphate; SUC, succinate; Xyl, xylose; X5P, xylulose 5-phosphate.

This study was initiated with the BL21p strain, which was previously developed to produce ethanol [21]. As indicated in Figure 1, it harbored a chromosomal copy of the *Zymomonas mobilis pdc* and *adhII* (the *pet* operon) responsible for the synthesis of ethanol. Catabolite repression was decoupled by inactivating *ptsG* in the strain. To facilitate glucose transport, the strain was modified by introducing the *Z. mobilis glf* gene. The genes involved in the pentose phosphate (PP) pathway were also enhanced to improve xylose metabolism. In addition, *ldhA*, *poxB*, *pta*, and *frdA* were eliminated to reduce the production of undesired by-products.

Ethanol has been reported to damage cell membranes and inhibit peptidoglycan synthesis [25,26]. Mutations in *E. coli* that confer high ethanol resistance are usually associated with modifications to cell membranes, including changes in fatty acid chain length and the trans-to-cis fatty acid ratio [27,28]. The inherent toxicity of ethanol limits the ethanol production in the BL21p strain. Therefore, adaptive evolution of the strain was conducted by gradually increasing the ethanol concentration. The lab-directed evolution was conducted for 100 days, and one resulting strain that survived in the presence of 40 g/L ethanol was isolated and designated BL21p-1. Cell characterization revealed that the specific growth rate of BL21p-1 was twice that of the BL21p strain in LB medium containing 30 g/L ethanol. It indicates that the evolved strain has improved its tolerance to ethanol.

**Figure 2:**
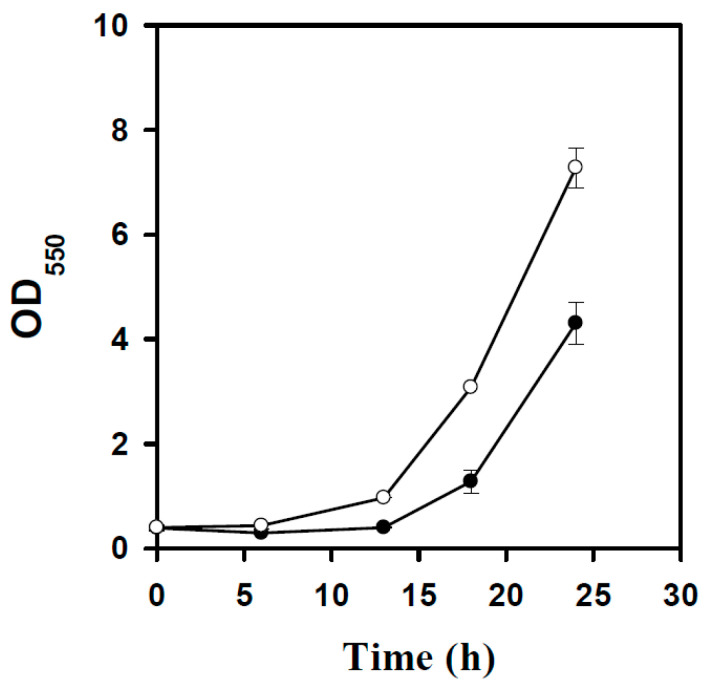
The growth profile of engineered strains on RSH. BL21p-1 and BL21-PP strains were cultured in shake flasks containing 40% (*v*/*v*) RSH, and their growth was monitored over time. The experiment was conducted in triplicate. The average data are presented. Keys: BL21p-1 strain (●); BL21-PP strain (○).

We next investigated the performance of the BL21p-1 strain. However, the BL21p-1 strain failed to grow on RSH. RSH was then diluted 2.5-fold (40% (*v*/*v*)) and used for cell culture. Figure 2 shows that the strain exhibited a prolonged lag phase, followed by slow growth. Note that RSH was prepared by pretreating rice straw with dilute acid at high temperatures [29]. This chemical treatment method produces toxic byproducts, primarily furan aldehydes, aliphatic acids, and phenolic compounds [30]. In particular, furfural and 5-hydroxymethylfurfural (5-HMF) are furan aldehydes that cause severe growth defects in cells [31]. Furfural is more toxic to living cells than 5-HMF and elicits the mutagenic interaction with DNA [32]. To detoxify the growth inhibitor, *E. coli* relies on the function of *yqhD*, which encodes an oxidoreductase that reduces furfural to furfuryl alcohol at the expense of NADPH [33]. The continued reduction in furfural reduces NADPH availability, thereby limiting NADPH-dependent biosynthetic pathways, such as the sulfate assimilation pathway [34]. This issue was addressed by increasing the intracellular NADPH level. The physiological function of the pentose phosphate (PP) pathway provides NADPH for biosynthesis. As illustrated in our previous study, the enhanced expression of *zwf* and *pgl* in the PP pathway increases NADPH availability in cells [35]. Therefore, *zwf* and *pgl* of the BL21p-1 strain were engineered to obtain the BL21-PP strain. In the presence of 40% (*v*/*v*) RSH, the engineered strain grew without any delay and outgrew the BL21p-1 strain (Figure 2).

### 3.2. Improvement of Furfural Tolerance

**Figure 3:**
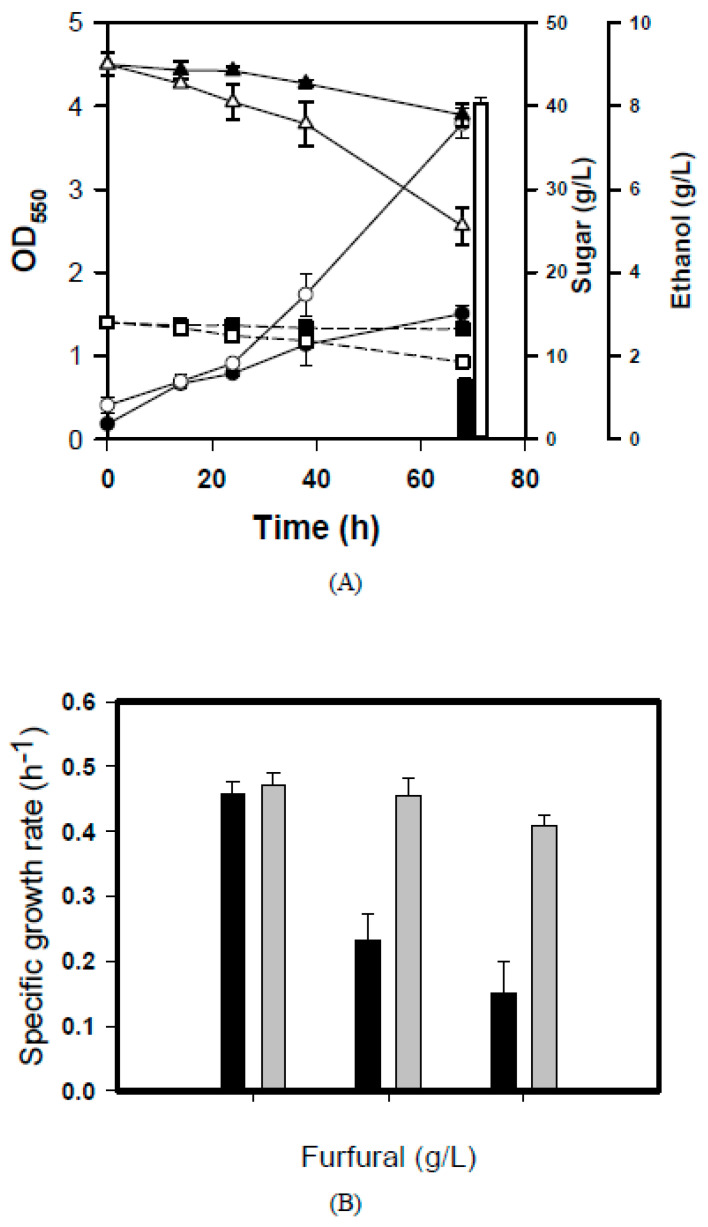
Fermentation profiles of engineered strains. The shake-flask culturing of BL21-PP and BL-E80 strains was performed using 70% (*v*/*v*) RSH. (**A**) The growth of the BL21-PP strain (●) and the BL-E80 strain (○) was followed by turbidimetric measurement. The sugar. consumption for the strains was measured over time. The ethanol production was ultimately determined. Keys: BL21-PP strain with glucose (▲), xylose (■), and ethanol (solid bar); BL-E80 strain with glucose (△), xylose (□), and ethanol (empty bar). (**B**) The furfural tolerance of strains was determined. Strains were grown on LB medium containing various concentrations of furfural, as indicated. Their specific growth rate was determined. Keys: BL21-PP strain (black bars); BL-E80 strain (gray bars). The experiments were conducted in triplicate. The average data are presented.

The BL21-PP strain was further characterized by using 70% (*v*/*v*) RSH. As shown in Figure 3A, the strain exhibited poor growth. This growth defect was attributed to elevated levels of toxic compounds in 70% RSH. The toxic mode of RSH likely results from the synergistic action of inhibitory compounds, which is elusive. It is challenging to target and optimize each epistatic gene involved in cellular detoxification. Accordingly, we decided to evolve the BL21-PP strain to ameliorate the pleiotropic effect of RSH toxicity. The adaptive evolution was carried out for 80 consecutive cycles, and the BL-E80 strain was isolated in the presence of 70% (*v*/*v*) RSH. As a result, BL-E80 exhibited a performance superior to the BL21-PP strain in terms of growth and sugar consumption (Figure 3A).

Furfural levels exceeding 5 mM are detrimental to cells [36]. The analysis showed that the BL21-PP strain was highly susceptible to furfural (Figure 3B). In contrast, the specific growth rate of the BL-E80 strain was less affected by furfural. Acetic acid is usually found in the hydrolysis of lignocellulosic biomass. It inhibits cell growth by depolarizing the cell membrane potential [37]. Interestingly, the BL-E80 strain tolerated more than 5 g/L of acetate, although the underlying mechanism remains unclear. It is recognized that three acid-resistance systems naturally exist in *E. coli* upon entry into the stationary phase [38]. In response to acid stress, *E. coli* modifies its membrane properties to alter proton permeability, induces regulatory proteins to prevent oxidative damage, and effectively adjusts ionic transporters to buffer the internal pH [39].

### 3.3. Production of Lignocellulosic Ethanol

**Figure 4:**
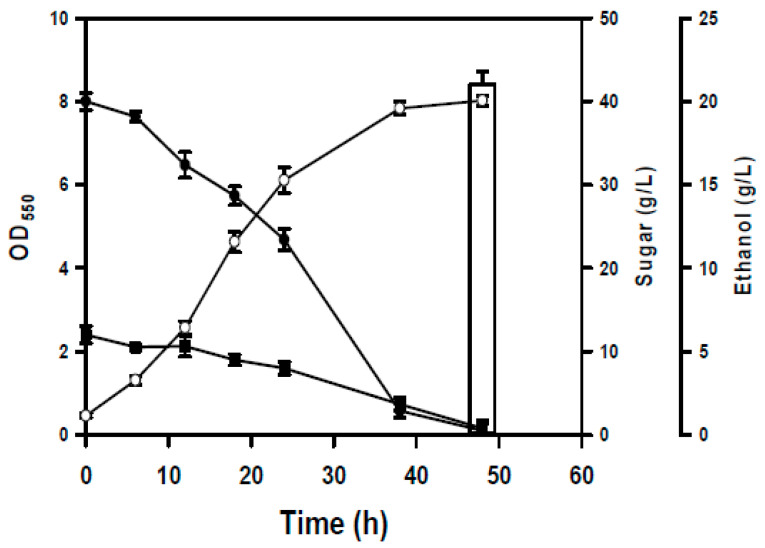
Fermentation profiles of the strain with engineered xylAB. The shake-flask culturing of the BL-E80a strain was performed using 70% (*v*/*v*) RSH. Cell growth (○) was followed by turbidimetric measurement. The sugar consumption for the strain was measured over time. The ethanol production was ultimately determined. Keys: glucose (●), xylose (■), and ethanol (bar). The experiments were conducted in triplicate. The average data are presented.

Figure 3A revealed that the BL-E80 strain metabolized xylose less efficiently than glucose. Note that this strain was derived from the BL21p strain equipped with the enhanced PP pathway (Figure 1). The BL21p strain enabled the equal utilization of pure glucose and pure xylose, as previously illustrated. This suggests that the impurities of RSH likely interfere with xylose metabolism in the strain. In *E. coli*, the catabolic pathway consisting of XylA and XylB is responsible for the conversion of xylose to xylulose 5-phosphate (X5P), an intermediate metabolite of the PP pathway. The xylose metabolism of the strain was then engineered by manipulating the endogenous *xylAB* operon, resulting in the BL-E80a strain. As shown in Figure 4, the modified strain outperformed the BL-E80 strain in terms of cell biomass and sugar consumption. The BL-E80a strain consumed all glucose and xylose, and its ethanol production reached 21 g/L at the end of the experiment. The result indicates that the improvement in the dissimilation pathway of xylose encourages the strain to utilize RSH.

### 3.4. Production of Lignocellulosic Ethanol and PHB

**Figure 5:**
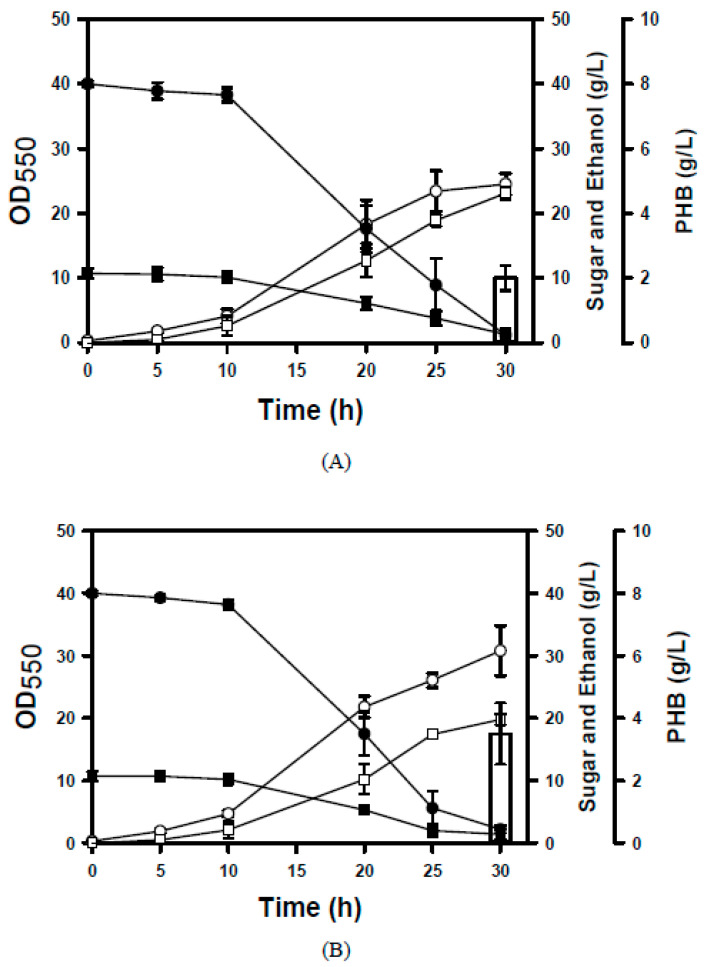
Lignocellulosic ethanol and PHB production for the engineered strains. (**A**) The shake-flask culturing of the E80-PHB1 strain was performed using 70% (*v*/*v*) RSH. The growth and sugar consumption were measured over time. The PHB production was ultimately determined. Keys: OD (○); glucose (●), xylose (■), ethanol (□), and PHB (bar). (**B**) The shake-flask culturing of the E80-PHB2 strain was performed similarly. Keys: OD (○); glucose (●), xylose (■), ethanol (□), and PHB (bar). The experiments were conducted in triplicate. The average data are presented.

The *phaCAB* operon encodes PHB synthase, β-ketothiolase, and NADPH-dependent acetoacetyl-CoA reductase, which are involved in the PHB synthesis pathway in *C. necator*. To produce PHB, the BL-E80a strain was engineered by recruiting the *phaCAB* operon of *C. necator*. This resulted in the E80-PHB1 strain harboring a genomic copy of the λP_L_-driven *phaCAB* operon. The PHB production is positively correlated with cell biomass [24] and requires acetyl-CoA as a precursor (Figure 1). The availability of oxygen favors the production of acetyl-CoA from pyruvate, which in turn leads to the synthesis of PHB. Therefore, aerobic culture of the engineered strain was used to produce the target products. The ethanol production in the E80-PHB1 strain accumulated over time and reached approximately 23 g/L at the end of the experiment (Figure 5A). This also led to the production of PHB at 2 g/L. Furthermore, a second copy of λP_L_-driven *phaCAB* was introduced into the E80-PHB1 strain, resulting in the E80-PHB2 strain. This strain was cultured in a manner similar to that of its parent strain. As shown in Figure 5B, the E80-PHB2 strain produced 19.8 g/L ethanol and 3.5 g/L PHB at the end of the experiment. Figure 1 shows that the ethanol and PHB synthesis pathways can compete for pyruvate. An increase in the gene copy number of *phaCAB* enhances the activity of the PHB synthesis pathway. Interestingly, ethanol production was slightly affected in PHB-producing strains compared with their parent strain (BL-E80a), which is incapable of producing PHB. The ethanol productivity consequently increases from 0.44 g/L/h (for BL-E80a) to 0.77 g/L/h (for E80-PHB1) and 0.66 g/L/h (for E80-PHB2). Overall, the ethanol yield on glucose and xylose reached 0.40–0.46 g/g, and the PHB content accounted for 27–38% of DCW. Byproducts, such as pyruvate and acetate, were below 2 g/L.

Recombinant *E. coli* has been previously developed for the production of lignocellulosic ethanol. Most research efforts were focused on the *pfl*- and *ldhA*-deficient FBR5 strain, which harbored the pLOI297 plasmid carrying the *pet* operon [40]. This strain exhibited a characteristic pattern of mixed sugar utilization, metabolizing glucose before xylose [41]. In a typical study, the FBR5 strain produced 21.9 g/L ethanol from non-abated wheat straw hydrolysate (WSH) within 90 h [42]. The productivity accounts for 0.24 g/L/h. The integration of simultaneous saccharification and fermentation (SSF) into the process essentially reduces the production cost of lignocellulosic ethanol. WSH at high solid loading was detoxified for the ethanol fermentation. The application of fed-batch SSF produced 41.6 g/L of lignocellulosic ethanol by the FBR5 strain within 120 h [43], yielding a productivity of 0.35 g/L/h. Another study reported that the plasmid-bearing FBR5 strain was stable and capable of producing 8.8–17.3 g/L of ethanol from WSH through continuous fermentation [44]. However, approximately 22.8% residual xylose and 1.4–3.1 g/L succinate were found in the fermentation broth. Few studies have examined the production of lignocellulosic PHB in *E. coli*. In a recent study, engineered *E. coli* was shown to produce PHB from corn stover hydrolysate (CSH) [45]. The maximum PHB production (3.2 g/L) was achieved at 96 h using a 2-fold-diluted CSH solution supplemented with 11.3 g/L peptone and an inoculum size of 14.2%. This results in the PHB productivity of 0.03 g/L/h. In contrast to these studies, this work aimed to engineer *E. coli* for the co-production of ethanol and PHB. The ethanol production and productivity ultimately reached 19.8–23 g/L and 0.66–0.77 g/L/h, respectively. The PHB production and productivity, respectively, accounted for 2–3.5 g/L and 0.07–0.12 g/L/h.

## 4. Conclusions

In this study, a producer strain was developed to produce ethanol from RSH. Without RSH detoxification, the engineered strain produced lignocellulosic ethanol by simultaneously utilizing glucose and xylose. Lignocellulosic ethanol and PHB were consequently co-produced by the strain following further reprogramming. Nevertheless, the economic viability of this production process would be acknowledged by increasing the production titer. Future work should focus on selectively producing ethanol and PHB during the fermentation course by integrating genetic engineering and fermentation strategies. Overall, this preliminary study indicates the potential application of this technology platform in lignocellulosic biorefineries.

## Figures and Tables

**Table 1:** Summary of strains and genetic materials used in this study.

Classification	Main Characteristics
Strain	
BL21p	Δ*ldhA*::ϕ80*attB*::λP_L_-*pet*, HK022*attB*::λP_L_-*glf*, Δ*poxB*::ϕ80*attB*::λP_L_-*rpe-talB*, P21*attB*::λP_L_-*rpiA-tktA*, Δ*ptsG*, Δ*pta*, Δ*frdA*
BL21p-1	evolved BL21p
BL21-PP	as BL21p-1, λP_L_-*zwf*, λP_L_-*pgl*
BL-E80	evolved BL21-PP
BL-E80a	as BL-E80, P*trc-xylAB*
E80-PHB1	as BL-E80a, λ*attB*::λP_L_-*phaCAB*
E80-PHB2	as E80-PHB1, ϕ80*attB*::λP_L_-*phaCAB*
Plasmid	
pPR-zwf	LE*-*kan*-RE*-λP_L_ flanked by two homologies of *zwf*
pSPL-atoD	LE*-*kan*-RE*-λP_L_ flanked by two homologies of *pgl*
pLam-Pha	λ*attP*, λP_L_-*phaCAB*
pPhi80-Pha	ϕ80*attP*, λP_L_-*phaCAB*
Primer	
Xyl-1	ggcttgcatattgaactccttgtgctcagtatcaccgccag
Xyl-2	tcatggtgtagggccttctgttccacacattatacgagcc
Xyl-3	aactcaaatgcgacatctgc
Xyl-4	aggagttcaatatgcaagcct
Xyl-5	ctgcgacatttgtgtttcttc
Xyl-6	cagaaggccctacaccatga

## Data Availability

The data presented in this study are available on request from the corresponding authors. The data are not publicly available due to protecting intellectual property.

## Abbreviations

The following abbreviations are used in this manuscript:

ATJ	Alcohol-to-jet
CSH	Corn stover hydrolysate
CSL	Corn steep liquor
DCW	Dry cell weight
5-HMF	5-Hydroxymethylfurfural
HPLC	High-performance liquid chromatography
PHA	Polyhydroxyalkanoate
PHB	Poly(3-hydroxybutyrate)
PP	Pentose phosphate
SSF	Simultaneous saccharification and fermentation
WSH	Wheat straw hydrolysate
